# Validation of the Vaccination Trust Indicator (VTI) in a multi-country survey of adult vaccination attitudes

**DOI:** 10.1371/journal.pgph.0001820

**Published:** 2023-04-12

**Authors:** Mallory K. Ellingson, Saad B. Omer, Nick Sevdalis, Angus Thomson

**Affiliations:** 1 Department of Epidemiology of Microbial Diseases, Yale School of Public Health, Yale University, New Haven, CT, United States of America; 2 Department of Internal Medicine, Infectious Disease, Yale School of Medicine, Yale University, New Haven, CT, United States of America; 3 Yale School of Nursing, Yale University, Orange, CT, United States of America; 4 Yale Institute for Global Health, Yale University, New Haven, CT, United States of America; 5 Center for Implementation Science, Health Service and Population Research Department, King’s College London, London, United Kingdom; 6 Department of Communication Studies & Global Health Communication Center, Indiana University School of Liberal Arts at IUPUI, El Paso, Texas, United States of America; University of Michigan, UNITED STATES

## Abstract

Improved uptake of adult vaccinations could substantially reduce the burden of infectious disease worldwide, however very few countries achieve high coverage of recommended adult vaccinations. Vaccine hesitancy is an important driver of low vaccine uptake among adults but no measure currently exists to capture general vaccination attitudes in this population accurately and efficiently. We utilize data from two surveys of adult vaccine attitudes and uptake conducted in fifteen countries to evaluate the Vaccination Trust Indicator (VTI). The VTI is a six-item measure intended to capture general vaccine attitudes. We utilized multivariable logistic regression to examine the association between VTI scores and self-reported receipt of the seasonal influenza vaccine, receipt of a tetanus toxoid-containing vaccine and intent to receive the flu vaccine in the next season. In the five countries with self-reported vaccine receipt data, we found that a ten-point increase in VTI score was associated with a 50% increase in odds of influenza vaccine receipt (OR = 1.55, 95% CI = 1.48, 1.62) and 25% increase in the odds of tetanus vaccine receipt (OR = 1.26, 95% CI = 1.21, 1.30). Strong associations between VTI score and vaccine receipt were found in each country except China. A strong association between VTI score and intent to receive the influenza vaccine was found in all fifteen countries. The VTI is a promising tool for assessing adult immunization attitudes with clear and immediate uses for immunization programs globally.

## Introduction

Vaccines are one of the greatest tools available to combat infectious disease. Childhood immunization programs have been a monumental public health success, preventing millions of deaths due to vaccine preventable diseases [1]. However, despite the availability of multiple vaccines to prevent common infectious diseases among adults, communicable disease continues to contribute substantially to global morbidity and mortality [2]. This burden is not shared equally, with the majority of infections and deaths due to vaccine preventable diseases among the elderly, individuals with underlying health conditions, displaced populations and those living in low- and middle-income countries [2, 3]. These patterns hold true for the SARS-CoV-2 pandemic as well, with the greatest burden of disease in individuals over 65 and those with underlying health conditions [4].

Improved vaccine uptake among adults could substantially decrease this burden of disease, particularly in light of the ongoing SARS-CoV-2 pandemic. Very few countries currently achieve routinely high vaccination rates with adult vaccines such as influenza, pneumococcal or tetanus-containing boosters, and the majority of countries have no adult vaccination program in place [5–9]. In many countries the influenza vaccine is only recommended or provided for high risk groups or is just not widely available [6, 9]. Tetanus vaccine recommendations vary even more widely, with many countries not recommending booster doses in adulthood [3]. The varied implementation of adult vaccine programs and the patchy availability of adult vaccinations globally contribute to low coverage but cannot entirely explain vaccine uptake among adults.

Vaccine hesitancy is defined by the World Health Organization (WHO) as a motivational state of being conflicted about, or opposed to, getting vaccinated and was named as one of the top ten health threats by the WHO in 2019 [10, 11]. For influenza vaccine, sociopsychological factors such as trust in vaccinations have been demonstrated to better explain past influenza vaccine behavior than demographic or socioeconomic factors [12]. As countries roll-out COVID-19 vaccination programs, hesitancy is emerging as a potential barrier to achieving the high vaccination rates required for herd immunity [13]. Multiple global surveys at the beginning of the pandemic suggested that on average nearly 30% of adults would not be likely to get a COVID-19 vaccine [13–16]. At present, very few countries have reached greater that 80% of the adult population completely vaccinated per the country’s protocol [17, 18]. However, none of these surveys have used consistent measures of vaccine hesitancy that have been validated against meaningful outcomes such as intention to vaccinate or actual vaccination behavior.

To understand and address vaccine hesitancy policymakers need to be able to measure and track vaccine confidence. This is important in efforts to increase uptake of existing vaccines, like the seasonal influenza and tetanus vaccines, but also vitally important in the introduction and dissemination of new vaccines or epidemics. Numerous scales exist to measure vaccine attitudes [19]; however, the current tools are limited in that the scales measure vaccine attitudes among specific to a population (i.e. parents of young children) [20–25] or for a specific vaccine (i.e. influenza or HPV) [12, 20, 22]. Most have only been tested and validated in high-income countries [26] and nearly all of the existing scales include ten or more items, limiting use as a “rapid diagnostic” tool.

In light of the need to rapidly and effectively measure vaccine attitudes in a general population, we sought to conduct a policy-guided validation of a Vaccination Trust Indicator (VTI). The goals of the VTI were to create a psychometrically viable yet easily administered tool that is relevant to a broad context of global settings, including low- and middle-income countries. There are many potential applications of such a tool, including rapid evaluation of general levels of vaccine confidence in individuals or populations at a given point in time, monitoring levels of general vaccine confidence in a population over time, or evaluating the effectiveness of interventions designed to improve in vaccine confidence among adults. In this study, we aim to validate the 6-item VTI among adults against self-reported influenza and tetanus vaccine receipt in five countries (US, UK, Mexico, France and China) and against intention to receive the influenza vaccine in the same five countries as well as ten additional high- and low-middle-income countries. Furthermore, we aim to determine clinically relevant cut-off levels for vaccine acceptance using the VTI and validate these against self-reported vaccine receipt in order to facilitate the use of this scale in intervention settings.

## Methods

### Participants and data sources

This validation analysis is a secondary analysis of existing data from two studies of adult vaccine attitudes that incorporated the VTI as part of the surveys. The first study was conducted at the end of the 2013/2014 influenza season in the United States, United Kingdom and France and at the end of the 2014/2015 influenza in China and Mexico. The second study was conducted in ten additional countries (Australia, India, Indonesia, Malaysia, Thailand, Vietnam, the Philippines, Taiwan, Singapore and Poland) at the end of the 2015/2016 influenza season (respectively in each region). The same survey methodology was utilized in each study and has been previously described elsewhere [12]. Briefly, stratified random sampling was utilized to obtain a nationally representative sample in each country. Participants were 18 years and older with at least a high school education and living in an urban setting. In both studies, a market research company was responsible for the survey administration (Double Helix and GfK, respectively for the two studies). A combination of online self-completion surveys and random digit dialing was used to reach the necessary proportion of 65+ and socioeconomic groups that may not have as much access to or familiarity with internet resources.

### Measures

The VTI is a 6-item scale intended to measure both trust and confidence in vaccines. The six items in the scale represent a small subset of a large 120-item vaccination attitudes survey [12]. The items in the VTI were selected based on preliminary analyses conducted during the 2013/2014 survey by the marketing company Double Helix which identified the unique items most highly correlated with the statement “I generally trust vaccination.” The items included in the scale represent core constructs previously demonstrated to be associated with vaccine confidence, including trust in key vaccine stakeholders, vaccine knowledge and vaccine effectiveness and self-efficacy (**Table 1**) [12]. Each item in the VTI utilizes an 11-point Likert scale (0–10), with lower scores representing disagreement with the statement and higher scores representing agreement. The score is an unweighted average of the participants’ responses to each question and scaled to a 100-point scale.

**Table 1:** Vaccine Trust Indicator items.

Thinking about vaccination in general, would you say you are personally….	0 = … strongly against vaccination … 10 = strongly for vaccination
I generally trust vaccine manufacturers or pharmaceutical companies	0 = strongly disagree …10 = strongly agree
I generally trust the {country specific public health authority} e.g. NHS (UK) / Department of Health (US) / National Health Authority (FR)	0 = strongly disagree … 10 = strongly agree
I understand how vaccination helps my body fight infectious disease	0 = strongly disagree …10 = strongly agree
I feel it is important that I get vaccinated	0 = strongly disagree …10 = strongly agree
Vaccination forms part of a healthy lifestyle	0 = strongly disagree … 10 = strongly agree

Both studies collected sociodemographic information including age, gender, education, race/ethnicity (where applicable) and self-reported health indicators. Survey items were adjusted to be culturally relevant in each country. In the first study, participants were asked if they had “received a flu vaccine (flu jab) in the past 6 months (this autumn/winter)?” and if they had “received a tetanus-containing adult booster in the last 10 years?” to assess receipt of the seasonal influenza and tetanus booster vaccines. Intent to receive the influenza vaccine in the next season was assessed in both studies using an 11-point Likert scale in response to the question “How likely are you to get the flu vaccine (flu jab) next winter (season)?” We created a bivariate intent measure where participants were defined as “intending to receive the flu vaccine” if their response was higher than the overall median intent and “not intending to receive the flu vaccine” if their response was lower than the overall median intent.

### Statistical analysis

**Fig 1:**
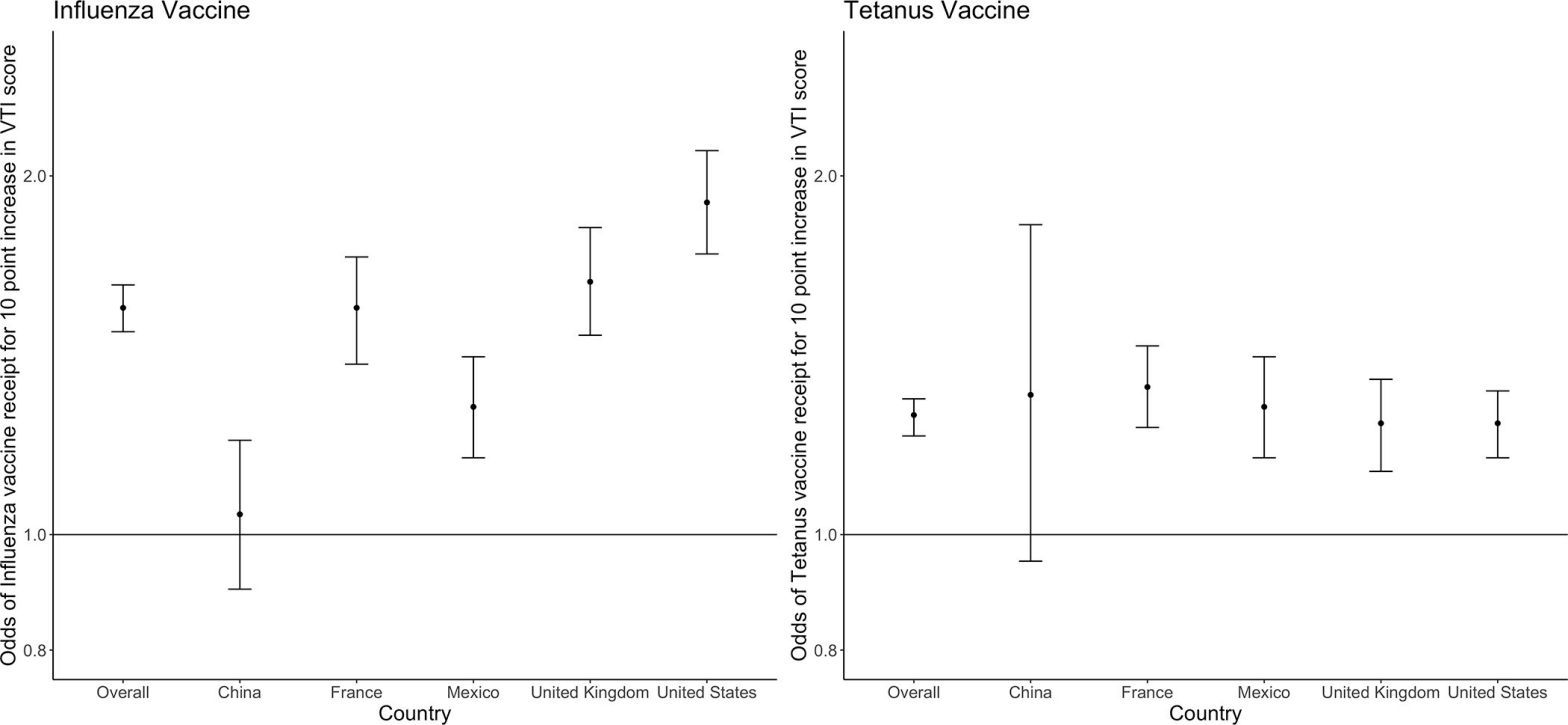
Odds of influenza and tetanus vaccine receipt for a ten-point increase in VTI score overall and by country.

Descriptive statistics and chi-square tests were calculated to explore variation in overall and country-specific socio-demographic characteristics, vaccine receipt and vaccine intent. We calculated the mean and standard deviation of the VTI overall and for each country. We visually assessed the distribution of VTI to identify natural change points and defined cut-offs based on this assessment (Fig 1) [22]. A VTI score lower than 40 was defined as “low trust in vaccines” a score between 40 and 70 was defined as “moderate trust in vaccines” and a score greater than 70 was defined as “high trust in vaccines.” The proportion of respondents in each category was assessed overall and within each country. To evaluate the usefulness of vaccine intent as a proxy for self-reported vaccine receipt in this validation analysis, we evaluated the correlation between bivariate vaccine receipt and the continuous measure of vaccine intent for the influenza vaccine in the five countries where data was available for both measures (US, UK, China, Mexico and France).

Logistic regression was used to calculate unadjusted odds ratios and 95% confidence intervals for the association between the mean scale scores and self-reported influenza and tetanus vaccine receipt overall and by country for those countries where vaccine receipt information was available. For the purposes of interpretation, the odds ratios were scaled such that the ratio can be interpreted as the “odds of vaccine receipt given a 10-point increase in VTI.” For all countries, the same approach was used to evaluate the association between mean scale scores and the bivariate measure of intent to receive the influenza vaccine in the next season. Both analyses were repeated adjusting for socio-demographic factors that may influence the relationship between the score and vaccine receipt including age, education, gender and presence of a chronic condition. An additional model was run to evaluate the overall association between VTI, vaccine receipt and vaccine intent adjusting for age, education, gender, presence of a chronic condition and country.

### Ethics statement

This study was a secondary analysis of de-identified data and received approval from the Emory University Institutional Review Board. The initial study received approval from the American Institutes for Research (USA), the Imperial College Research Ethics Committee (UK), and waivers to approval from the French Commission nationale de l’informatique et des libertès and Comités de protection des personnes. All participants were informed about the nature of the study and provided consent. Sanofi Pasteur provided an educational grant to support the data collection for the initial study and gave the authors access to this data for the current analyses.

## Results

The overall study sample contained 9,809 participants, with responses varying from 400–850 within each country. Fifteen countries were included, although self-reported influenza and tetanus vaccine receipt information was only available for five of them. Among the five countries where vaccine receipt data was available (China, France, Mexico, the United Kingdom and the United States), approximately 18% of the sample was over the age of 65. Across all fifteen countries included, the study sample was predominantly between the ages of 23–55 and mostly college-educated (or equivalent). Males and females were equally represented and approximately 44% of the population reported at least one chronic health condition (**Table 2; Table A in S1 Table**).

**Table 2:** Socio-demographic characteristics of the study population and vaccine receipt.

	Overall (N = 9809)		Received Influenza Vaccine in Past Six Months			Received Tetanus-Toxoid Containing Vaccine in Past Ten Years		
	N	%	N	%	p-value	N	%	p-value*
**Total**			1333	31%		1905	45%	
**Country** **					<0.001			<0.001
China	879		102	12%		19	2%	
France	848		198	23%		584	69%	
Mexico	850		310	37%		520	61%	
United Kingdom	847		316	37%		295	35%	
United States	846		407	48%		487	58%	
**Age**					<0.001			0.11
18–22 years	878	9%	50	22%		89	40%	
23–35	2971	30%	314	28%		481	43%	
36–55	3573	36%	399	26%		682	44%	
56+	2387	24%	570	41%		653	47%	
**Age**					<0.001			0.07
<65			964	27%		1547	44%	
Over 65			369	49%		358	48%	
**Gender**					0.78			0.19
Female	4834	49%	692	31%		1016	46%	
Male	4975	51%	641	31%		889	44%	
**Chronic Health Condition** *					<0.001			<0.001
Yes	4333	44%	700	45%		828	54%	
No	5476	56%	633	23%		1077	39%	
**Education**					<0.001			<0.001
No formal education/No education reported	149	2%	70	52%		48	36%	
Less than high school or high school equivalent	952	10%	162	30%		204	38%	
High school diploma or equivalent	1634	17%	346	29%		545	45%	
Some higher education	1451	15%	72	42%		92	54%	
University degree	4011	41%	385	28%		518	37%	
Graduate degree	1276	13%	217	42%		310	60%	
Other	142	1%	42	30%		73	51%	

*P-values calculated using chi-squared test

**Vaccine receipt data not available for Australia, Indonesia, India, Malaysia, Philippines, Singapore, Thailand, Taiwan or Vietnam, therefore percentages do not sum to 1005

***Individuals were categorized as having a chronic health condition if they reported one or more of the following: diabetes, high or low blood pressure, chronic obstructive pulmonary disease, chronic kidney condition, chronic inflammatory bowel condition, chronic heart condition, a weakened immune system, cancer, a chronic neurological condition, a chronic liver condition or another chronic condition.

In the five countries where vaccine receipt information was available, 1,333 (31%) of participants reported receiving the influenza vaccine in the last influenza season and 1,905 (45%) reported receiving a tetanus-toxoid containing vaccine within the past ten years (**Table 2**). Reported vaccine receipt varied widely by country with nearly 50% of respondents reporting having received the influenza vaccine in the United States versus 12% reporting receipt in China. Influenza vaccine receipt also varied by age and presences of a chronic disease, with individuals over the age of 65 and individuals with a chronic disease having the highest reported vaccine receipt (49% and 45% respectively).

The overall average VTI score was 73.8 (SD = 18.5) (**Table 3**). The Philippines had the highest average score (83.8, SD = 14.4) and France and China had the lowest VTI scores (64.3, SD = 19.8 and 14.9, respectively). Average VTI scores did not vary widely by any socio-demographic characteristics. Most participants were categorized as having high trust in vaccines (61%) or moderate trust in vaccines (33%) (**Table 3**). Only 5% of participants were categorized as having low trust in vaccines. The proportion of respondents in each VTI category also varied widely by country. In the United States, 13% of respondents had low trust in vaccines compared to only 1% in India, Malaysia, Thailand, the Philippines and Vietnam. Over 80% of participants were categorized as having high trust in vaccines in Mexico, the Philippines and Indonesia.

**Table 3:** Vaccine Acceptance Index Scores by country and sociodemographic characteristics.

	Vaccine Acceptance Index Score		Vaccine Acceptance Index Categories*					
			Low		Moderate		High	
	Mean	SD	N	%	N	%	N	%
**Overall**	73.8	18.5	531	5%	3256	33%	6022	61%
**Country**								
Australia	77.9	18.6	18	5%	96	24%	386	72%
China	64.3	14.9	65	7%	498	57%	316	36%
France	64.3	19.8	100	12%	404	48%	344	41%
Indonesia	81.1	14.9	11	2%	107	17%	497	81%
India	81.5	14.5	7	1%	126	21%	468	78%
Malaysia	75.3	15.2	6	1%	140	35%	259	64%
Mexico	81.9	17.6	31	4%	135	16%	684	80%
Philippines	83.8	14.4	7	1%	118	16%	630	83%
Poland	65.7	19.6	78	11%	321	45%	308	44%
Singapore	68.9	15.7	18	5%	197	49%	185	46%
Thailand	80.6	13.4	6	1%	122	22%	424	77%
Taiwan	65.4	15.5	23	5%	290	58%	191	38%
United Kingdom	73	17.7	50	6%	285	34%	512	60%
United States	68.3	22.5	107	13%	284	34%	455	54%
Vietnam	80.1	13.8	4	1%	133	22%	463	77%
**Received Influenza Vaccine** **								
Yes	79.7	15.1	22	2%	318	24%	993	74%
No	66.1	20.2	331	11%	1288	44%	1318	45%
Received Tetanus Vaccine***								
Yes	75	18.9	102	5%	577	30%	1226	64%
No	66.6	19.7	251	11%	1029	44%	1085	46%

*Vaccine acceptance categories are defined as follows: < = 40 = low vaccine acceptance, 41–70 = moderate vaccine acceptance, 71 or greater = high vaccine acceptance

**Defined as self-reported receipt of the seasonal influenza vaccine in the past six months, only available for China, France, Mexico, United States and United Kingdom

Overall, a ten-point increase in VTI score was associated with 55% increased odds of influenza vaccine receipt (OR = 1.55, 95% CI = 1.48, 1.62) and 26% increased odds of tetanus vaccine receipt (OR = 1.26, 95% CI = 1.21, 1.30) (**Fig 1, Table C in S1 Table**). However, in China specifically there was no meaningful association between VTI score and vaccine receipt (Flu: OR = 1.04, 95% CI = 0.90, 1.20; Tetanus: OR = 1.31, 95% CI = 0.95, 1.82). The overall and country-specific relationships were similar when adjusted for the relevant covariates (**Table D in S1 Table**).

In the 65+ population, a ten-point increase in VTI score was associated with a 65% increased odds of reported influenza vaccine receipt (OR = 1.65, 95% CI = 1.50, 1.83) and a 16% increased odds of tetanus vaccine receipt (OR = 1.16, 95% CI = 1.08, 1.26). The relationship was similar in the under-65 population (flu: OR = 1.54, 95% CI = 1.46, 1.62; tetanus: OR = 1.28, 95% CI = 1.23, 1.33).

**Fig 2:**
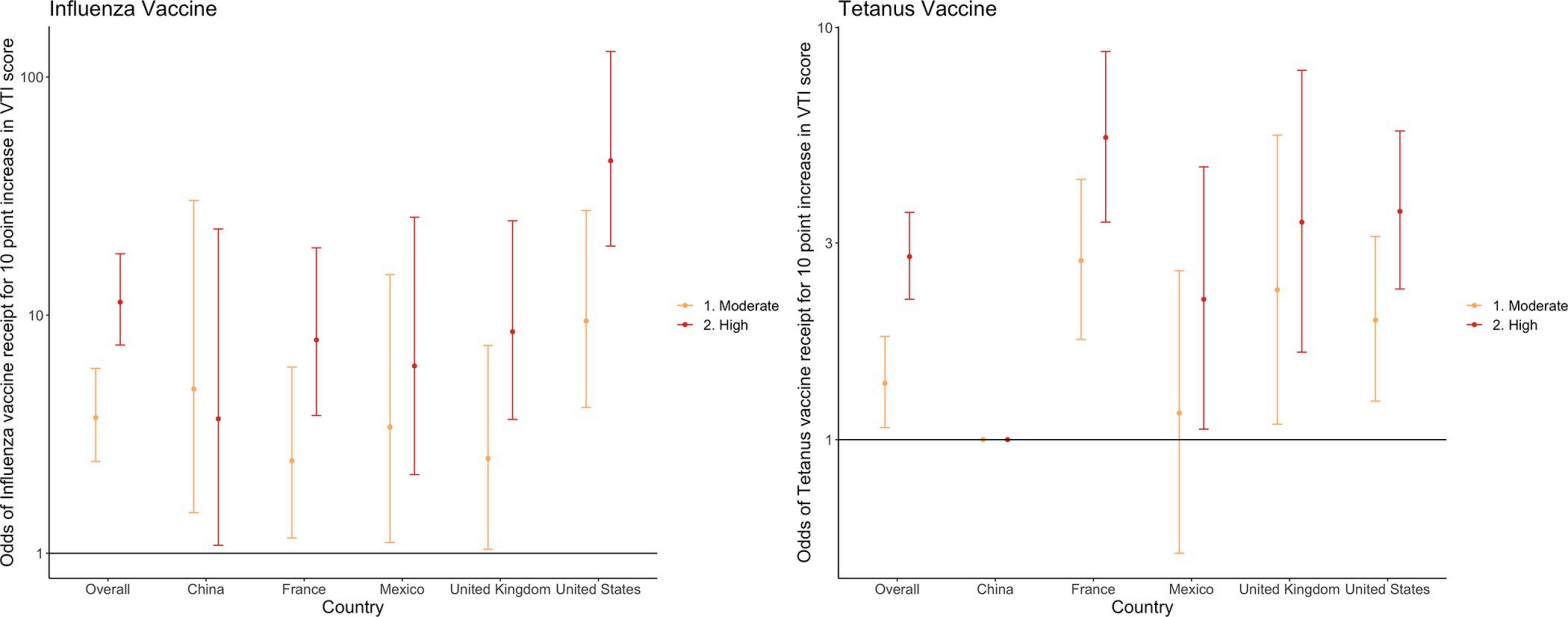
Odds of influenza and tetanus vaccine receipt by VTI score category overall and by country *reference group = low trust in vaccines.

Individuals whose score indicated moderate trust in vaccines were 3.71 times more likely to have received the influenza vaccine (95% CI = 2.43, 5.98) and 1.37 times more likely to have received the tetanus vaccine (95% CI = 1.07, 1.78) (**Fig 2, Table C in S1 Table**). Those with high trust in vaccines were 11.34 times more likely to receive the influenza vaccine (95% CI = 7.49, 18.1) and 2.78 times more likely to receive the tetanus vaccine (95% CI = 2.19, 3.56). In Mexico, there was no meaningful relationship between moderate trust and tetanus vaccine receipt (OR = 1.16, 95% CI = 0.53, 2.57), however high trust in vaccines was associated with a two-fold increase in odds of tetanus vaccine receipt (OR = 2.19, 95% = 1.06, 4.59). The overall and country-specific associations were similar when adjusted for the relevant covariates (**Table D in S1 Table**).

Among the 65+ population specifically, the relationship was similar to the overall population. Individuals whose score indicated moderate trust in vaccines were 3.64 times more likely to have received the influenza vaccine (95% CI = 1.75, 8.56). Those with high trust in vaccines were nearly twelve times more likely to have received the influenza vaccine (OR = 11.9, 95% CI = 5.84, 27.63). The relationship was similar among the under 65 population (moderate: OR = 4.0, 95% CI = 2.39, 7.31; high: OR = 12.27, 95% CI = 7.39, 22.16).

Among the fifteen countries where self-reported influenza vaccine intent information was available, approximately 50% of participants reported intent to receive the influenza vaccine in the next flu season (51%; **Table 4**). Intent to receive the influenza vaccine also varied widely by country, with only 31% of participants in Poland reporting intent to receive the influenza vaccine in the next season whereas nearly 70% of participants in Mexico reported intending to receive the flu vaccine (67%). In the five countries where vaccine receipt data was available, 93% of those who reported receiving the influenza vaccine in the past flu season also reported that they intended to get the influenza vaccine in the next flu season (1236/1333, p < 0.001).

**Table 4:** Unadjusted associations between Vaccine Acceptance Index score and intent to receive the influenza vaccine in the next influenza season*.

	Intent to Receive Influenza Vaccine*		Vaccine Acceptance Index Score**			Vaccine Acceptance Index Category***	
	N	%	OR	95% CI		OR	95% CI
**Overall**	5019	51%	1.74	1.70, 1.80	*moderate*	4.63	3.44, 6.38
					*high*	19.81	14.78, 27.19
**Country**							
Australia	223	56%	1.64	1.43, 1.90	*moderate*	****	****
					*high*	****	****
China	434	49%	1.33	1.21, 1.46	*moderate*	1.27	0.75, 2.19
					*high*	2.87	1.66, 5.04
France	261	31%	1.7	1.54, 1.89	*moderate*	6.98	2.82, 23.23
					*high*	22.38	9.10, 74.31
Indonesia	364	59%	1.79	1.56, 2.07	*moderate*	4.27	0.77, 79.93
					*high*	19.94	3.77, 367.50
India	312	52%	1.64	1.44, 1.88	*moderate*	****	****
					*high*	****	****
Malaysia	186	46%	1.75	1.5, 2.08	*moderate*	****	****
					*high*	****	****
Mexico	571	67%	1.84	1.66, 2.07	*moderate*	4.51	1.49, 19.58
					*high*	30.57	10.65, 128.99
Philippines	489	65%	1.78	1.58, 2.02	*moderate*	****	****
					*high*	****	****
Poland	218	31%	1.83	1.63, 2.06	*moderate*	6.11	2.18, 25.47
					*high*	24.36	8.84, 100.88
Singapore	136	34%	1.67	1.42, 1.99	*moderate*	****	****
					*high*	****	****
Thailand	367	66%	1.69	1.45, 1.98	*moderate*	****	****
					*high*	****	****
Taiwan	232	46%	2.32	1.98, 2.77	*moderate*	9.58	1.97, 172.97
					*high*	65.54	13.22, 1189.41
United Kingdom	371	44%	1.7	1.54, 1.89	*moderate*	2.67	1.17, 7.20
					*high*	9.5	4.29, 25.23
United States	471	56%	2.34	2.09, 2.65	*moderate*	14	5.65, 46.66
					*high*	107.29	43.55, 357.46
Vietnam	384	64%	1.51	1.32, 1.72	*moderate*	2.12	0.26, 43.38
					*high*	7.29	0.92, 148.07

*Participants were asked to rate how likely they were to receive the influenza vaccine in the next season on a scale of 0 to 10. Intent to receive the vaccine was defined as a score greater than the overall median intent

**For ease of interpretation, Vaccine Acceptance Index score was categorized into a ten-point scale. Therefore the odds ratio can be interpreted as the increased odds of intent to vaccinate for a ten-point increase in VAI score

***Low vaccine acceptance used as reference group

****Small cell counts, model would not converge

In general, a ten-point increase in VTI score was associated with increasing odds of intent to receive the influenza vaccine in the next flu season, although the strength of the association varied by country (**Table 4**). When individuals were placed in vaccine acceptance categories (VTI ≤ 40, low vaccine acceptance; 41–70, moderate vaccine acceptance; ≥ 71, high vaccine acceptance), those with moderate trust in vaccination were over four times more likely to have the intention to receive the influenza vaccine than those with low trust in vaccines (OR = 4.63, 95% CI = 3.44, 6.38). Individuals with high trust in vaccines were nearly 20 times more likely to intend to get the flu vaccine (OR = 19.81, 95% CI = 14.78, 27.19).

## Discussion

Using data from multi-country surveys of vaccine attitudes and receipt among adults we developed a 6-item measure (VTI). Scores from the VTI were strongly associated with self-reported influenza and Tdap vaccine receipt in all but one country surveyed. Furthermore, VTI score was also highly associated with intent to receive the seasonal influenza vaccine in all countries surveyed. In our categorization of VTI scores, we found that individuals who were categorized as having a “high” level of trust in vaccines were two to three times more likely to report having received the influenza or tetanus, diphtheria and acellular pertussis vaccine (Tdap) than those who were categorized as having “low” trust in vaccines. These results indicate that the Vaccine Trust Index has the potential to be a valid and accurate measure of adult vaccine attitudes in both individuals and populations.

Trust is a foundational component of vaccine acceptance; trust not just in the vaccines, but also in the providers of the vaccines–healthcare workers, public health authorities and governments [27–31]. We previously showed that in the US, France and UK datasets that trust in vaccine manufacturers, health authorities and providers was correlated with vaccination intentions [32]. A lack of trust in the government or in pharmaceutical companies was associated with uptake of the MMR vaccine among mothers of young children and trust in modern medicine was correlated with uptake of the influenza vaccine among the elderly [27]. Institutional mistrust was associated with decreased vaccination rates in children across 21 countries in Africa [33]. Presently, trust in authorities and vaccine stakeholders is emerging as a key determinant of public acceptance of COVID-19 vaccines [34], likely moderated by the level of transparency and consistency in the implementation of COVID-19 control measures by health authorities [15]. We may therefore expect that post-COVID-19 the VTI may be an even stronger indicator of vaccine intentions and uptake.

Across all five countries with self-reported vaccine receipt data, we found that a ten-point increase in VTI score was associated with a 50% increase in odds of self-reported influenza vaccine receipt and 25% increase in the odds of self-reported tetanus vaccine receipt. This strength of association is comparable to the few existing, validated measurements of vaccine attitudes. In Gilkey et al.’s validation of their Vaccine Confidence Scale they found that an increase in mean scale score was associated with a 20% to 50% increase in odds of provider-verified vaccine receipt [22]. Similarly, Frew et al. found an approximately 10% increase in odds of vaccine receipt of each recommended childhood vaccine for each increase in their measure of vaccine confidence among parents in the US [20].

The strength of the association between VTI scores and self-reported vaccine receipt varied by country, with a null or weak association between VTI score and both influenza and Tdap vaccine receipt in China. We believe this is likely a reflection of differing adult immunization policies by country. At the time that this survey was conducted there was no universal recommendation of the influenza vaccine for the general adult population in China, although there has been growing emphasis on influenza vaccination in China [35]. The consistent association between VTI score and vaccine receipt in many other countries suggests that the VTI may be widely applicable in a variety of settings. However, there is room for further refinement to improve generalizability. Furthermore, although we did find a consistently strong correlation between “high” VTI scores and vaccine receipt compared to “low” VTI scores, the association between scores categorized as “moderate” and vaccine receipt was also inconsistent. Further investigation is needed to explore the utility of the categorizations of the VTI as defined in this study.

Our study is subject to limitations; first, the surveys were conducted across three different influenza seasons and fifteen different countries. Although the same methodology was utilized for all surveys, it is possible that responses, particularly for the influenza vaccine, were influenced by the different influenza seasons limiting the interpretation of the overall association between VTI score and vaccine receipt. However, all within-country surveys were conducted at the same time by the same investigators, highlighting the validity of the country-specific results. Second, in the third round of surveys conducted among adults in Poland and the nine countries in the Asia-Pacific region investigators did not ascertain self-reported vaccine receipt due to the inconsistency in national influenza immunization programs and availability of adult vaccinations. Therefore, in these eleven countries we were only able to validate VTI score against intent to receive the influenza vaccine. However, we did find in our analysis that among respondents in the five countries where intent and receipt information was available for the influenza vaccine more that 90% of respondents who indicated that they received the influenza vaccine in the past season also intended to receive the vaccine in the next season. Lastly, vaccine receipt is based on self-reported information rather than provider verified records of immunization history. However, at least in the United States studies have demonstrated that self-report is a good measure of immunization history among adults [36, 37].

To understand and address vaccine hesitancy, vaccination programs need to be able to measure and track vaccine confidence. This is important in efforts to increase uptake of existing vaccines, like the seasonal influenza and tetanus vaccines, but has been shown to be vitally important for COVID-19 vaccination programs. The VTI is not vaccine-specific and can be utilized to measure attitudes about any adult vaccines. Our analyses have demonstrated that the VTI is valid measure of vaccine attitudes in non-Western, non-high-income countries. In the context of adult immunizations, it is also important to ensure that any measurement of vaccine attitudes is valid among the most at risk or target populations, which is most commonly adults over the age of 65. VTI score was associated with vaccine receipt among the 65+ population in our study. Lastly, nearly all of the existing scales include ten or more items, limiting use as a “rapid diagnostic” tool. The VTI utilizes six items and can be used to rapidly categorize individuals into “high,” “moderate” or “low” levels of trust in vaccines, making it very useful in the intervention setting.

The VTI is a promising tool for assessing adult immunization attitudes with clear and immediate uses for immunization programs globally. This is particularly important in light of the COVID-19 pandemic and the international COVAX initiative to ensure equitable access to a COVID-19 vaccine. Countries should consider monitoring trust in vaccination among adults with the VTI over time to inform country readiness and demand generation activities for all vaccination programs, including COVID-19.

## Supporting information

S1 Table **A.** Sociodemographic characteristics of study population by country. **B.** Vaccine Trust Index scores by sociodemographic characteristics of the study population. **C.** Unadjusted association between Vaccine Acceptance Index Score and Vaccine Receipt. **D.** Adjusted association between Vaccine Acceptance Index Score and Vaccine Receipt.
(XLSX)
